# Stress-induced ECM alteration modulates cellular microRNAs that feedback to readjust the extracellular environment and cell behavior

**DOI:** 10.3389/fgene.2013.00305

**Published:** 2013-12-31

**Authors:** Evgeniia V. Edeleva, Halyna R. Shcherbata

**Affiliations:** Max Planck Research Group for Gene Expression and Signaling, Max Planck Institute for Biophysical ChemistryGöttingen, Germany

**Keywords:** miRNAs, extrinsic stress, ECM composition, bi-directional signal transduction, dystroglycan

## Abstract

The extracellular environment is a complex entity comprising of the extracellular matrix (ECM) and regulatory molecules. It is highly dynamic and under cell-extrinsic stress, transmits the stressed organism’s state to each individual ECM-connected cell. microRNAs (miRNAs) are regulatory molecules involved in virtually all the processes in the cell, especially under stress. In this review, we analyse how miRNA expression is regulated downstream of various signal transduction pathways induced by changes in the extracellular environment. In particular, we focus on the muscular dystrophy-associated cell adhesion molecule dystroglycan capable of signal transduction. Then we show how exactly the same miRNAs feedback to regulate the extracellular environment. The ultimate goal of this bi-directional signal transduction process is to change cell behavior under cell-extrinsic stress in order to respond to it accordingly.

Each individual organism in its lifetime has to cope with multiple kinds of stress that occur when body’s homeostasis is shifted from its optimal state. Development, puberty, changing environmental conditions, injuries, diseases, and aging – all influence the organism as a whole no matter which cell or system was the first to encounter the stressor. Organism’s systemic reaction to stress is achieved because cells are not isolated from each other. Rather, they communicate through direct cell–cell contacts or via the extracellular matrix (ECM).

In this review, in the beginning we introduce the ECM and the cell-extrinsic stress-induced signaling molecules. We define the extracellular environment, focusing on how it can encode for stress signals. We then discuss how a cell can read the encoded signals, focusing on cell adhesion molecule dystroglycan (Dg) capable of outside-in signal transduction. In the last two parts of the review, we show how a cell can change levels of intracellular microRNAs (miRNAs) in response to outside stress signals, and how these miRNAs can then modulate the cell behavior by targeting components of the extracellular environment.

## ECM AS A RESERVOIR FOR CELL-EXTRINSIC STRESS-INDUCED SIGNALING MOLECULES

The ECM is composed of three major types of structural components: insoluble collagen fibers, viscous proteoglycans, and soluble multiadhesive proteins. Examples of multiadhesive proteins are laminin and fibronectin that connect collagens and proteoglycans to the cell receptors. Metabolic enzymes responsible for the restructuring of the ECM, like matrix metalloproteinases (MMPs) and tissue inhibitors of metalloproteinases (TIMPs), can be deposited outside the cell (Yu and Woessner, 2000; Yu et al., 2000) and thus can be considered as the fourth, enzymatic, component of the ECM (**Table 1**).

**Table 1:** The components of the extracellular environment, cellular sensory apparatus, and miRNAs, mentioned in the review.

**Extracellular environment**				
ECM components				
Structural				Enzymatic
Collagen fibers	Proteoglycans		Multiadhesive proteins	Metabolic enzymes
	HSPG:	SLRP:	- Laminins	- MMPs
	- Perlecan	- Decorin	- Fibronectin	- TIMPs
	- Agrin	- Biglycan		
		- Fibromodulin	

**Cell-extrinsic stress-induced molecules**				

**Cytokines**		**Hormones**	**Extracellular miRNAs**	
Growth factors:	Other:	Androgen	- Packaged into lipid-based carriers
- TGFs	- TNF-α	Ecdysone – *Drosophila* thyroid	- Bound by RNA-binding proteins
- BMPs	- IL-7	hormone homolog	
- HGF	- IL-6	*Drosophila* insulin-like peptide	
- PDGF			
- VEGF -			
FGFs			
- EGF			

**Cellular sensory apparatus**				
**Cell adhesion molecules**		**Cell signaling receptors**		
- Integrins		- Cytokine receptors
- Dystroglycan		- Receptor tyrosine kinases
- CD44		- TGF-β receptors
- Fasciclin II		- Nuclear receptors
		- Receptor phosphotyrosine phosphatases

**Outside-in and inside-out messengers: miRNAs**				
**Dependent on the extracellular environment**		**Modulating the extracellular environment**		
*miR-34 family*		*miR-34a*
*miR-205*		*miR-205*
*miR-133/miR-1*		*miR-133*
*miR-29 family*		*miR-29*
*dme-miR-252, dme-miR-980, dme-miR-956*		
*miR-21*		*miR-21*
*miR-132*		*miR-132*
*miR-125 family*		*miR-125*
*dme-let-7-Complex*		*dme-let-7*
*dme-miR-14, dme-miR-8*		*dme-miR-14, dme-miR-8*
*miR-143/miR-145*		*miR-143*
*miR-133/208/499*		*miR-133*

The three-dimensional net of ECM components acts as a reservoir and a scaffold for a diversity of regulatory molecules: cytokines (including various growth factors and interleukins), hormones, and extracellular miRNAs. These molecules constitute the cell-extrinsic stress-induced signaling components, as they warn different cells of the organism about changes in the environmental conditions such as injuries, pathogens, and other external stressors that endanger the organism’s homeostasis and welfare.

## THE EXTRACELLULAR ENVIRONMENT

Extracellular environment can be defined as a combination of the ECM and the extracellular regulatory molecules. Specific composition of the ECM of each ECM-connected cell defines the types of regulatory molecules found in close proximity to the cell. For example, different types of cytokines can be bound to different types of ECM proteins, contributing to where the cytokine exerts its function in mediating a specific cell fate or activity, as we now discuss.

### COLLAGEN-BINDING CYTOKINES

Procollagen of type IIB is synthesized and deposited into the ECM by differentiated chondrocytes. Immature chondrogenic progenitors synthesize the other splice variant of procollagen – type IIA that has an additional cysteine-rich domain. This domain was shown to bind growth factors, such as transforming growth factor-β1 (TGF-β1), bone morphogenetic proteins bone morphogenetic protein-2 (BMP-2) and BMP-4 (Zhu et al., 1999; Larraín et al., 2000). Hence, the type of ECM procollagen defines the presence of differentiation factors and thus cell’s responsiveness to them.

Types of collagen deposited by cells also impose spatial constraints on the biological activity of platelet-derived and hepatocyte growth factors (PDGF and HGF; Somasundaram and Schuppan, 1996; Schuppan et al., 1998). PDGF and HGF trigger mitosis in mesenchymal cells and hepatocytes respectively. Thus, the ECM collagenous composition serves as an important clue for growth factors binding to the appropriate target cell types.

### FIBRONECTIN-BINDING CYTOKINES

Cell-to-ECM adhesion protein fibronectin can also modulate effects of various signaling molecules, as for example of vascular endothelial growth factor (VEGF) that promotes migration and proliferation of endothelial cells. Fibronectin has separate cell-binding and VEGF-binding domains, and only when it binds VEGF and a cell simultaneously, the VEGF stimulation is significantly enhanced (Wijelath et al., 2006). Thus, the degree of endothelial cell response to VEGF can be regulated by the ECM composition.

Another domain of fibronectin was shown to interact with tumor necrosis factor-α (TNF-α) – initiator and regulator of inflammatory reactions. When bound to fibronectin, TNF-α enhances adhesion of activated immune cells to this glycoprotein, suggesting that ECM-bound TNF-α may recruit and direct immune cells to the sites of inflammation (Hershkoviz et al., 1994). In addition, interleukin-7 (IL-7) has been shown to modulate adhesive properties of immune cells in the context of the ECM composition. IL-7 binding to fibronectin augments adhesion of resting T-cells to fibronectin (Ariel et al., 1997). Hence, the ECM environment in which immune reactions take place modulates effects of cytokines on immune cells.

### HSPG-BINDING CYTOKINES

Heparan sulfate proteoglycans (HSPGs) are cell surface and ECM molecules composed of a protein core to which heparan sulfate chains are attached. Sulfate chains can bind basic fibroblast growth factors (FGF) for storage and protection from proteolytic degradation (Saksela et al., 1988). Bound FGFs can be released in bioactive form by partial proteolysis of the protein core or through digestion of heparan sulfate moieties (Ishai-Michaeli et al., 1990; Saksela and Rifkin, 1990). However, in order to interact with their cell receptors basic FGFs have to be bound by heparan sulfate chains (Rapraeger et al., 1991; Yayon et al., 1991).

Perlecan and agrin are HSPGs involved in modulation of FGFs signaling in the processes of bone formation and neurite outgrowth respectively. Perlecan secreted by chondrocytes localizes to the growth plate of the developing long bones; FGF18 bound to perlecan enhances FGF receptor 3 signaling to control proper cartilage/bone transition zone formation (Chuang et al., 2010). Agrin in neuronal basal laminae binds FGF2 and probably enhances FGF2 affinity to its cellular receptor resulting in enhancement of FGF2 neurite outgrowth stimulation (Kim et al., 2003).

Small leucine-rich proteoglycans (SLRPs) represent a different type of proteoglycan-growth factor interactions. The core proteins of SLRPs decorin, biglycan and fibromodulin were shown to bind to isoforms of TGF-β (Hildebrand et al., 1994), sequestering TGF-β into the ECM for signaling.

### EXTRACELLULAR miRNAs

In addition to cytokines, the extracellular environment also contains hormones and miRNAs. Recent studies have suggested that these extracellular miRNAs can act as regulatory molecules. miRNAs are small non-coding regulatory RNA molecules. In most cases, they act as negative regulators of protein translation by binding to the 3′UTRs of the target mRNA molecules, subjecting them for silencing or degradation (He and Hannon, 2004). miRNAs are encoded by the genome of each organism. They undergo the maturation process first in the nucleus and then in the cytoplasm. Extracellular miRNAs are expressed by a cell but they are secreted into the extracellular environment to act on other cells. They are different from intracellular miRNAs, which act on the same cell that have expressed the miRNA (reviewed in Rayner and Hennessy, 2013).

Components of the ECM and ECM-tethered and free cell-extrinsic stress-induced signaling molecules (cytokines, hormones, and extracellular miRNAs) together constitute the extracellular environment, surrounding each individual cell (summarized in **Table 1**). This environment is highly dynamic and changes in response to different stimuli. The profile of the signaling molecules may be altered or the composition of the ECM may change, which will also bring a change in the quantity and distribution of certain ECM-associated signaling molecules. Thus, the extracellular environment represents the state of the organism, translated into the language that each individual cell can understand: in the language of cell-extrinsic stress-induced signaling molecules.

## HOW DO EXTRACELLULAR SIGNALS REGULATE miRNA EXPRESSION

In general, the cell can interpret the extracellular signals because it has various receptors that enable the ECM-bound cell to communicate with the outside world by modulating signal transduction. There are two main types of receptors: (1) cell signaling receptors responsible for binding and transducing signals from cell-extrinsic stress-induced signaling molecules, and (2) cell adhesion molecules that bind and respond to the ECM components.

**FIGURE 1:**
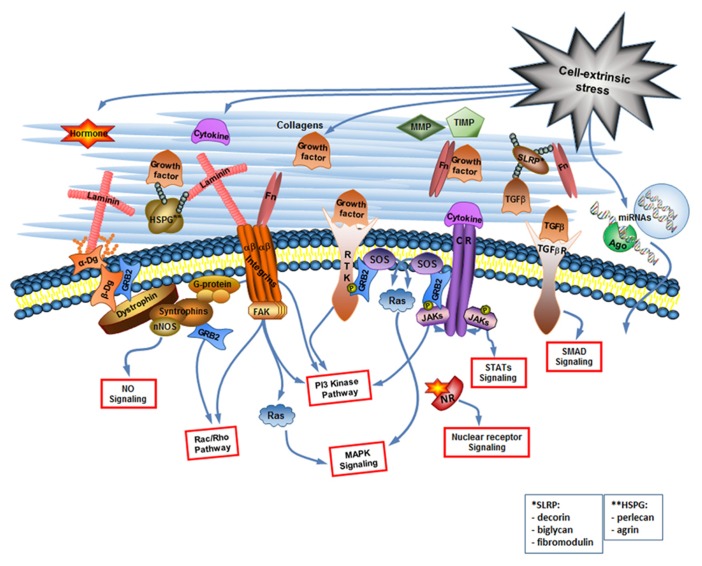
**The cell-extrinsic stress signaling brings about the changes in the extracellular environment that are read by the cellular sensory apparatus.** The extracellular environment is a complex but organized entity composed of structural and enzymatic ECM proteins [collagens, proteoglycans1, multiadhesive proteins like laminin and fibronectin (Fn), MMPs and TIMPs] and cell-extrinsic stress-induced signaling molecules including cytokines (considering growth factors as a type of cytokine), hormones, and extracellular miRNAs. The cellular sensory apparatus includes the ECM receptors and receptors for signaling molecules. Integrins and dystroglycan (Dg) are the ECM receptors. They do not only connect cell to its matrix but are also involved in the cell signaling. Multiple receptors for signaling molecules [including receptor tyrosine kinases (RTK), cytokine receptors (CR), TGF-β receptors, and nuclear receptors (NR)] transduce changes in the extracellular environment into the various signaling pathways inside the cell. Extracellular miRNAs can probably be taken up by the cell via yet unknown mechanism. NO, nitric oxide, Ago, argonaute.

Cell signaling receptors, such as cytokine receptors (CR), receptor tyrosine kinases (RTK), TGF-β receptors, nuclear receptors (NRs), and receptor phosphotyrosine phosphatases among others, bind growth factors, interleukins, and other cytokines, steroid and protein hormones. Each type of receptor activates specific signaling pathways inside the cell but often pathways activated by different types of receptors overlap. For example, the Ras-mitogen-activated protein (MAP) kinase pathway, and the PI-3 kinase pathway are common to more than one type of receptor (**Figure 1**). Since many miRNAs are encoded by their own genes, the pathways activated by cell signaling receptors can regulate expression of miRNA-coding genes as well as of protein-coding genes.

Most prominent cell adhesion molecules are the proteins of the integrin family that bind to collagen, perlecan, laminin, and fibronectin. Intracellular integrin signaling can act either through modulation of signaling from growth receptors or through direct activation of intracellular signaling events. Directly activated by integrins are the MAP kinase pathway, the PI3 kinase (PI3K) pathway, and the small GTPases of the Rho family (Keely et al., 1998; Giancotti, 1999; **Figure 1**). Although integrin signaling has a potential to regulate miRNA expression profile (Gerson et al., 2012), we would like to focus here on another cell adhesion molecule Dg capable of regulating miRNA expression. Dg is a part of the Dystrophin Glycoprotein Complex, associated with a group of fatal inherited diseases muscular dystrophies (Campbell, 1995; Cohn, 2005; Goddeeris et al., 2013). No cure exists for these neuromuscular degenerative diseases, and we believe that better understanding of Dg role in cell-ECM communication will aid in the development of future therapeutics.

## DYSTROGLYCAN AS A CELL ADHESION MOLECULE

Dystroglycan is a major non-integrin ECM binding receptor. α- and β-Dgs are translated in mammals from the same mRNA transcript and are then separated by posttranslational cleavage and modifications to form a membrane complex with extracellular α-Dg tightly bound to transmembrane β-Dg (Barresi and Campbell, 2006; **Figure 1**).

α-Dystroglycan can bind to different ECM proteins: laminin, agrin, and perlecan in muscle, and neurolexin in brain (Barresi and Campbell, 2006). Importance of the α-Dg association with the ECM is highlighted by the experimental data that mice with mutation in the gene encoding Dg do not develop into adults due to improper formation of the early embryonic basement membrane (Williamson et al., 1997). In Dg-null murine muscle, α7B integrin receptor subunit is selectively upregulated suggesting that certain types of integrins can partially compensate for the absence of Dg in basement membrane assembly and maintenance (Côté et al., 2002).

Transmembrane β-Dg binds inside the cell to dystrophin in skeletal muscles and to alternative proteins of the dystrophin locus or to an autosomal homolog of dystrophin, utrophin, in non-muscle tissues (Barresi and Campbell, 2006). β-Dg also binds the adaptor protein growth factor receptor-bound protein 2 (Grb2) both in muscle and in brain (Yang et al., 1995). Dystrophin localizes to the plasma membrane another type of adaptor proteins – syntrophins with different syntrophin isoforms being expressed in muscle and neuronal tissues (Bhat et al., 2013).

The interaction partners of Dg and dystrophin, as well as the Dg–dystrophin signaling are extensively studied in mammalian muscle cell culture and in muscle tissue, as well as on the *Drosophila* muscular dystrophy model (Shcherbata et al., 2007). In the genetic interaction screen on *Drosophila*, we identified new muscle specific partners of Dg and dystrophin, of which some are implicated in mechanical and stress-induced pathways (Kucherenko et al., 2011). Loss of Dg or dystrophin in *Drosophila* resulted in altered cellular levels of reactive oxygen species, suggesting the function of these proteins in regulation of homeostasis (Marrone et al., 2011b). In muscle cell culture, phosphorylation of syntrophins due to laminin-1 binding to α-Dg was shown to increase the association between syntrophins and Grb2, resulting in the initiation of Rac1 signaling (Zhou et al., 2006). In skeletal muscle, syntrophins also bind heterotrimeric G-protein. This binding together with laminin attachment to Dg complex is necessary for activation of the PI3K/Akt signaling pathway (Xiong et al., 2009). Interestingly, recent data also links the Dg–dystrophin signaling via syntrophins to the expression of miRNAs in muscle progenitors. Syntrophins can localize neuronal nitric oxide synthase (nNOS) to the muscle cell sarcolemma leading to the production of nitric oxide as a second messenger that nitrosylates histone deacetylase 2 (HDAC2) influencing expression of certain genes, including miRNAs, important for muscle cell differentiation and maintenance (Cacchiarelli et al., 2010).

The nervous system expression of Dg together with dystrophin and syntrophins suggests the role for Dg in the nervous tissue. Importance of the cell-to-ECM adhesion via Dg in the nervous system is supported by the fact that mice with astrocytes-specific deletion of Dg show discontinuities in the pial surface basal lamina (Moore et al., 2002). In the genetic interaction screen on *Drosophila* brain, we showed that Dg and dystrophin interact with proteins involved in actin cytoskeleton remodeling, which is essential for cell homeostasis (Marrone et al., 2011a). Moreover, defects in the Dg glycosylation that disrupt its association with the ECM cause numerous human diseases, such as muscle-eye-brain disease, Walker–Warburg syndrome, forms of congenital muscular dystrophies, symptoms of which include prominent neurological abnormalities (Cohn, 2005).

Hence, integrins, Dg, and a variety of signaling receptors for the cell-extrinsic stress-induced signaling molecules constitute the major cellular sensory apparatus, allowing the cell to read information encoded by the extracellular environment (**Table 1**; **Figure 1**). Signaling pathways activated by the cellular sensory apparatus have a potential to regulate intracellular miRNA expression, examples of which we discuss in the next section.

## miRNAs DEPENDENT ON THE EXTRACELLULAR ENVIRONMENT

The importance of extracellular environment for regulation of miRNA expression profile was highlighted in the experiments with Matrigel. Matrigel is a 3D cell culture medium composed of the protein mixture secreted by the mouse sarcoma cells, resembling the basement membrane (Kleinman and Martin, 2005). Human cancer cells cultured on the Matrigel have a significantly different miRNA expression profile compared to cells cultured on plastic (Price et al., 2012). Hence, miRNA cellular levels depend on the composition of the extracellular environment. In the similar experimental setup, it was shown that p53 expression levels and nuclear localization are enhanced in human cells cultured on Matrigel (Li et al., 2003). Taking into account that certain miRNAs – *miR-34* family members and *miR-205*– can be directly regulated by p53 (Shi et al., 2008; Piovan et al., 2012), these results again suggest that cellular miRNA profile can change in response to the extracellular environmental composition.

miRNA expression profile can be regulated in the ECM-dependent manner at the transcription level through (a) regulation of epigenetic marks or (b) through regulation of transcription factors’ activity. Additionally, cellular miRNA expression profile can be altered through direct incorporation of extracellular miRNAs.

### EPIGENETIC MODIFICATIONS

miRNA expression can be regulated through changes in the chromatin modifications. For example, inhibition of histone deacetylases (HDACs) was shown to alter miRNA levels in breast cancer cell line (Scott et al., 2006). Many pathways can lead to HDACs inhibition. One of the signaling mechanisms via HDAC2 inhibition can be suggested to modulate levels of specific miRNAs in muscle progenitor cells depending on the extracellular environment. This pathway involves dystrophin and nNOS localization to plasma membrane by syntrophins. nNOS causes nitric oxide production, S-nitrosylation and subsequent inhibition of HDAC2 that directly controls expression of *miR-133/miR-1* and *miR-29* (Cacchiarelli et al., 2010). Since dystrophin is tightly bound to Dg, and Dg is an important ECM binding molecule, it can be hypothesized that Dg–dystrophin–syntrophin–nNOS–HDAC2 pathway can be modulated from the extracellular space in response to stimuli to alter miRNA levels.

Though this pathway was described on muscle progenitor cells, it may be important for tuning miRNA expression in non-muscle tissues as well. Dg, dystrophin and syntrophin isoforms were shown to be expressed in non-muscle tissues, like in brain, liver, and kidney (Tinsley et al., 1994; Bhat et al., 2013). Moreover, we have identified on whole-fly *Drosophila* RNA extracts a group of Dg and dystrophin-dependent stress-response miRNAs. Levels of these miRNAs change under temperature stress in *wild type* flies. On the contrary, in flies with no functional Dg or dystrophin, no change in the expression of these miRNAs is observed. At least three of such Dg- and dystrophin-dependent stress-response *Drosophila* miRNAs –*miR-252*, *miR-980,* and *miR-956*– depend also on the levels of syntrophin 1 that is a *Drosophila* homolog of mammalian syntrophins capable of nNOS localization (Alessi et al., 2006; Marrone et al., 2012). Since *miR-252*, *miR-980,* and *miR-956* are suggested to have a nervous system expression, the Dg–dystrophin–syntrophin–NOS–HDAC pathway may exist additionally in non-muscle tissues and contribute to the miRNA expression changes under stress.

### MODIFICATIONS VIA TRANSCRIPTION FACTORS

The change in the miRNA transcription due to external stimulation can occur because of the modulation of the availability and activity of certain transcription factors. For example, TGF-β signaling acts via direct activation of cytosolic Smad transcription factors, and *miR-29* was shown in human cell culture to be regulated downstream of TGF-β2 pathway (Luna et al., 2011). Interestingly, this same miRNA is involved in the muscle cell differentiation and maintenance program and, as discussed above, is controlled by the Dg–dystrophin–syntrophin–nNOS–HDAC2 pathway.

Various immune-response cytokines act via direct activation of signal transducer and activator of transcription (STAT) factors where STAT dimer is translocated into the nucleus upon phosphorylation by JAK receptors in response to extracellular signaling. *miR-21* – a tumor suppressor miRNA associated with many types of cancer – has been shown to be regulated by this signaling pathway since the promoter of the *miR-21* gene can be directly bound by STAT3 transcription factor (Löffler et al., 2007).

Many cytokines via Ras-mitogen-activated protein kinase (MAPK) signaling can activate cAMP-response element binding protein (CREB), which binds to promoters and regulates transcription of various genes. The expression of one of the CREB-activated genes in rat neuronal cells *miR-132* is turned on in response to neurotrophins stimulation (Vo et al., 2005). Growth factor signaling activates several pathways and kinase cascades, including MAPK signaling cascade, leading to activation of transcription factors specific to the cell type and external stimulus. For example, *miR-125* expression is reduced in human lung cancer cell culture due to epidermal growth factor (EGF) stimulation, though it is not yet clear which transcription factors are involved in this response (Wang et al., 2009).

Many steroid hormones belong to the NR family. Inactive NRs are cytoplasmic. Upon hormone binding, they translocate into the nucleus and serve as transcription factors. miRNAs directly activated by NRs exist. For example, human *miR-125b-2* contains a functional androgen-responsive element upstream of its gene locus (Shi et al., 2007). In *Drosophila*, *miR-125* belongs to an evolutionary conserved *let-7-Complex* of three miRNAs, *miR-100*, *let-7*, and *miR-125* (Pasquinelli et al., 2000). Ecdysteroid hormone signaling was shown to upregulate transcription of *let-7-Complex* but repress transcription of other *Drosophila* miRNAs *miR-14* and *miR-8* (Varghese and Cohen, 2007; Garbuzov and Tatar, 2010; Chawla and Sokol, 2012; Jin et al., 2012; Kucherenko et al., 2012). Such NR-responsive miRNAs can react very fast to hormone stimulation without multiple intermediate activation steps required.

### DIRECT INPUT FROM THE ECM

In addition to the ECM-dependent modulation of signaling pathways that leads to changes in the intracellular miRNA profile, extracellular miRNAs can be directly incorporated by cells. miRNAs can be prominently secreted from certain tissues, transported via blood by lipid- or protein-carriers and can then be taken up by recipient cells in different tissues (Rayner and Hennessy, 2013). For example, in human endothelial cells atheroprotective shear stress significantly upregulated expression of *miR-143/miR-145* cluster. *miR-143/miR-145* were then found enriched in exosomes of endothelial cells, secreted by them and taken up by co-cultured smooth muscle cells. This prevented smooth muscle cells from de-differentiation (Hergenreider et al., 2012). Hence, in the organism *miR-143/miR-145* secreted from the endothelial cells in response to atheroprotective shear stress may travel via blood to the smooth muscle cells and protect them from malfunction.

miRNAs can be secreted from inflamed tissues, signaling that a possible threat for the whole organism exists. Analysis of plasma from patients allowed to suggest that *miR-133/208/499* are secreted from myocardium following acute myocardial infarction (AMI) and circulate in blood packaged into exosomes or microvesicles (Corsten et al., 2010; De Rosa et al., 2011; Olivieri et al., 2013).

**FIGURE 2:**
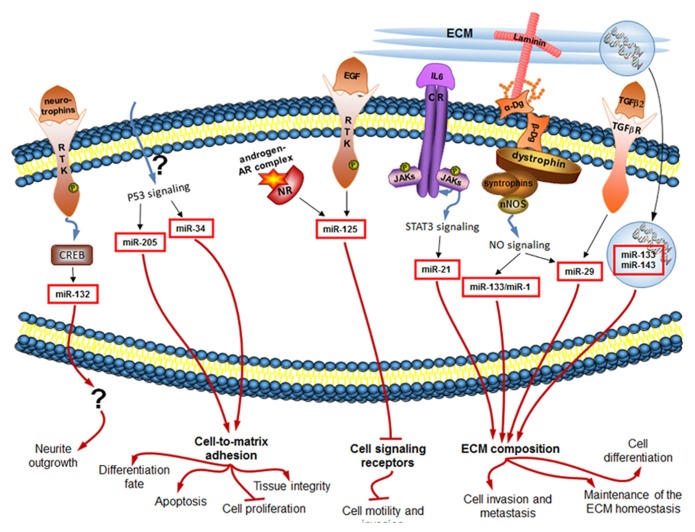
**miRNAs regulated downstream of signaling induced by changes in the extracellular environment (black arrows) influence in turn the ECM composition and the cellular sensory apparatus, and ultimately the cell behavior (red arrows).** Only mammalian miRNAs and pathways are shown.

miRNAs take part in virtually all critical cellular processes (He and Hannon, 2004), ranging from stem cell division, maintenance and differentiation (Hatfield et al., 2005; Qi et al., 2009; Mathieu and Ruohola-Baker, 2013) to aging of the organism (Inukai and Slack, 2013). We have now summarized some evidence that the constantly tuned and adjusted miRNA profile of cells is regulated downstream of many classical ECM-connected pathways, as well as of a newly discovered ECM-connected Dg–dystrophin–syntrophin–nNOS–HDAC2 pathway (**Figure 2**).

## ALTERED miRNA PROFILE CHANGES CELL BEHAVIOR

miRNAs differentially expressed in response to ECM-dependent signaling may have various functions in the cell. Interestingly, a proportion of those ECM-regulated miRNAs was shown to feedback to alter the ECM composition or the cellular sensory apparatus (**Figure 2**). In this section, we would like to highlight how each of the introduced in the previous section miRNAs modulates cell behavior by acting either on the components of the extracellular environment or on the cell adhesion molecules/cell signaling receptors (**Table 1**).

### MODULATION OF CELL BEHAVIOR VIA EXTRACELLULAR ENVIRONMENT

The ECM is a remarkably complex still organized entity. Its careful regulation is essential for proper functioning of cells and subsequently tissues, organs and the whole organism. miRNAs can regulate the ECM composition. For example, the above discussed TGF-β2 signaling-dependent *miR-29* family members were shown to play a role in the maintenance of the ECM homeostasis. Expression of *miR-29* members in human cell culture resulted in significant reduction of the ECM components, such as laminin, fibronectin, collagen I, collagen IV, and SPRC (secreted protein, acidic, and rich in cysteine; Villarreal et al., 2011). *miR-133*, which is secreted from diseased myocardium following AMI, was shown in the experiments in rats and in human cell culture to directly target 3′UTR of the pro-α1 chains of type I collagen, changing the ECM properties of the recipient tissues (Castoldi et al., 2012). Interestingly, miR-133 is also one of the miRNAs activated by the Dg–dystrophin–syntrophin–nNOS–HDAC2 pathway in muscle progenitor cells, and it is known there to inhibit cell proliferation via inhibition of the serum response factor, and to induce cell differentiation via HDAC4 inhibition [as summarized in (Marrone and Shcherbata, 2011)].

Fibronectin is a high-molecular weight ECM glycoprotein that binds integrin receptors modulating their signaling activity thus playing an important role in cell adhesion, growth, differentiation, and migration. Fibronectin mRNA can be targeted by miRNAs. In human cell culture experiments *miR-143* was shown to directly target the 3′UTR of the fibronectin type III domain containing protein 3B – molecule that regulates cell motility. By repressing fibronectin expression, high levels of *miR-143* induce invasive and metastatic behavior of liver tumors in mice (Zhang et al., 2009). It is interesting to note, that *miR-143* is one of the miRNAs found in the extracellular space packaged into exosomes (Hergenreider et al., 2012). Since *miR-143* is an onco-miRNA, cancer cells may communicate and promote each other’s metastatic and invasive properties via secretion of this circulating miRNA.

For proper ECM maintenance, availability and activity of ECM modulating enzymes MMPs and TIMPs are of utmost significance. miRNAs were shown to control their expression. Elevated levels of the IL-6 dependent oncogenic *miR-21* were shown to inhibit MMP inhibitors, promoting MMP activity, cancer invasion, and metastasis of glioblastoma in mouse model (Gabriely et al., 2008). Another study on human tissues suggested that *miR-21* induces inhibition of TIMP3, and additionally of programmed cell death 4 protein, accounting for increased invasiveness and reduced apoptosis in cholangiocarcinomas (Selaru et al., 2009).

Signaling molecules are a part of the extracellular environment, and miRNA signaling can target such molecules. For example, expression of the insulin-like peptide in *Drosophila*, secreted by insulin-producing cells in the fly brain and involved in control of energy homeostasis, is regulated by *miR-14*. It was recently reported that *miR-14* directly targets in insulin-producing cells a negative regulator of *insulin-like peptide* gene expression. Thus, ecdysteroid-dependent *miR-14* provides a link between steroid hormone signaling and insulin secretion to allow nutrient-independent insulin-production control (Varghese et al., 2010). Another *Drosophila* miRNA *miR-8*, repressed by ecdysteroid signaling, is involved in innate immune homeostasis control. It keeps antimicrobial peptides’ expression by fly innate immune system organ, fat body, at low basal level. Hence, in pathogen-free flies *miR-8* is suggested to keep immune system from firing and thus to prevent autoimmune reactions (Choi and Hyun, 2012).

### MODULATION OF CELL BEHAVIOR VIA CELL ADHESION MOLECULES AND SIGNALING RECEPTORS

As important as the extracellular environment is for cell behavior, proper cell functioning depends also on appropriate cell-to-ECM attachment and signaling. miRNAs play a role in regulation of cell adhesion and signaling as well. For example, it was shown that silencing of the p53-induced *miR-205* in human prostatic cell line reduces secretion of all laminin-332 subunits and integrin-β4 – critical cell-to-ECM adhesion molecules in control of tissue integrity. Hence, loss of *miR-205* in prostate cancer results in basement membrane discontinuities (Guess and Quaranta, 2009; Gandellini et al., 2012). Another p53-induced *miR-34a* can directly target CD44 in human cancer cell lines. CD44 is a glycoprotein expressed on cell surfaces and capable of ECM binding, playing a role in cell migration and adhesion. *miR-34a*-induced decrease in CD44 levels was shown to inhibit prostate cancer regeneration and metastasis (Liu et al., 2011). In another work on mice, directed delivery of *miR-34a* to cancer cells with nanovector resulted in induced apoptosis, decreased proliferation, and ultimately inhibition of tumor growth (Pramanik et al., 2011).

Proper cell adhesion is important not only for stable differentiated state of the cell as opposed to de-differentiation during cancerogenesis, but also for the early development and initial differentiation of the cell. Neuronal differentiation is a good model for emphasizing the importance of cell adhesion in the complex process of nervous system development. When neurons differentiate, they send their axons to defined places and establish contacts with specific cells and ECM components due to tightly controlled cell adhesion process. miRNAs fine-tune this process. For example, in rat cortical neurons *miR-132* expression in response to stimulation with neurotrophins results in neurite outgrowth (Vo et al., 2005). Ecdysteroid induced miRNA *let-7* in the developing *Drosophila* brain controls via a cytokine-dependent transcription factor expression levels of the neural cell adhesion molecule Fasciclin II, ultimately regulating neurons’ differentiation fate (Kucherenko et al., 2012; discussed in Kucherenko and Shcherbata, 2013). Interestingly, in the differentiated neuron the target of *let-7* is a negative regulator of ecdysteroid signaling. Thus, *let-7* is involved in the positive feedback loop to enhance its own expression level.

*miR-125* gives an example of a miRNA that regulates expression of cell signaling receptors. In human cancer cell line, *miR-125* members are inhibited due to EGF stimulation (Wang et al., 2009). Interestingly, *miR-125* overexpression results in the reduction of the transcript and protein levels of the EGF receptors themselves, significantly reducing cell motility and invasion (Scott et al., 2007). Hence, *miR-125* is also involved in the positive feedback loop down-regulating its own inhibitor, and leading to inhibition of certain properties of cancerous cells.

## CONCLUDING REMARKS

The extracellular environment is a complex system of ECM components and signaling molecules. It undergoes constant rearrangements following different kinds of stress. Cells have developed a sensitive apparatus to respond to the changes of the extracellular environment by tuning the expression of genes, including the miRNA-coding genes. Interestingly, some of those miRNAs feedback to affect the ECM and cellular sensory apparatus with the ultimate goal being, to change the cell behavior. This further widens the idea of the “dynamic reciprocation” between the cell and its extracellular environment under stress (Bissell et al., 1982).

**FIGURE 3:**
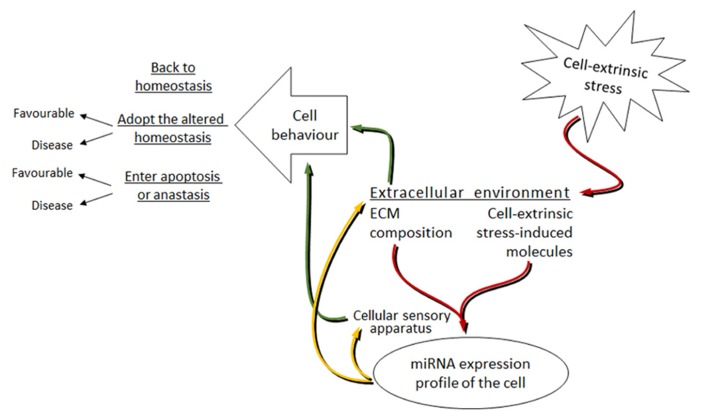
**Proposed model of the miRNAs involvement in the bi-directional signal transduction between the cell and extracellular environment under cell-extrinsic stress.** Red arrows: cell-extrinsic stress changes the extracellular environment of cells in the organism. The ECM composition and the cell-extrinsic stress-induced molecules, as parts of the altered extracellular environment, signal via the cellular sensory apparatus to change the miRNA expression profile of cells. Yellow arrows: altered miRNA levels target the cellular sensory apparatus and the extracellular environment. Green arrows: this leads to the changes in cell behavior. The mode of cell behavior is different dependent on the strength and length of the applied cell-extrinsic stress.

Although it is the extreme cases of miRNAs influencing cell behavior (like invasion, metastasis and apoptosis) that are mostly studied, we can hypothesize that under mild stress, miRNAs together with other effector molecules in an affected cell coordinate their work to return the organism to its initial homeostasis. The stronger and longer the stress is – the harder it is for the cell to constantly shift back to its initial state. Then, a new altered homeostasis is obtained. The new homeostasis represents the stress-induced state of the cell and though in most cases favorable, it can sometimes lead to cell transformations resulting in, for example, cancer. Under extreme levels of stress, a cell may enter apoptosis or turn into anastasis (**Figure 3**).

In this review, we focused on the ECM bound cells and on their reaction toward the cell-extrinsic stress. The general picture is much more complex. Many cells do not have contacts to the ECM and organize into three-dimensional structures due to multiple cell-to-cell contacts. In addition to cell-extrinsic stress, cell-intrinsic stress, independent from extracellular environmental signaling, can lead to the changes in cell behavior. In those cases, the cellular transcriptome, including the miRNA profile, will be the one to change first. It may then be translated into the changes in the cell behavior and at the same time into the changes in the ECM composition and cell adhesion. Both may again change the cellular transcriptome and miRNA profile, and ultimately fine-tune the cell behavior.

With the growing field of miRNA research, further studies will identify new ECM-dependent pathways or will prove that canonical pathways regulate miRNA expression. As miRNAs seem to be ubiquitous to all cellular processes, for many of them a role in the regulation of the ECM composition and the cellular sensory apparatus will be attributed. This will firmly consolidate the role of miRNAs in the bi-directional signal transduction process between the cell and its exterior.

## Conflict of Interest Statement

The authors declare that the research was conducted in the absence of any commercial or financial relationships that could be construed as a potential conflict of interest.
